# Influence of Connexin40 on the renal myogenic response in murine afferent arterioles

**DOI:** 10.14814/phy2.12416

**Published:** 2015-05-25

**Authors:** Jens Christian B Jacobsen, Charlotte M Sorensen

**Affiliations:** Department of Biomedical Sciences, Division of Renal and Vascular Physiology, University of CopenhagenCopenhagen, Denmark

**Keywords:** Afferent arteriole, autoregulation, connexin, myogenic

## Abstract

Renal autoregulation consists of two main mechanisms; the myogenic response and the tubuloglomerular feedback mechanism (TGF). Increases in renal perfusion pressure activate both mechanisms causing a reduction in diameter of the afferent arteriole (AA) resulting in stabilization of the glomerular pressure. It has previously been shown that connexin-40 (Cx40) is essential in the renal autoregulation and mediates the TGF mechanism. The aim of this study was to characterize the myogenic properties of the AA in wild-type and connexin-40 knockout (Cx40KO) mice using both in situ diameter measurements and modeling. We hypothesized that absence of Cx40 would not *per se* affect myogenic properties as Cx40 is expressed primarily in the endothelium and as the myogenic response is known to be present also in isolated, endothelium-denuded vessels. Methods used were the isolated perfused juxtamedullary nephron preparation to allow diameter measurements of the AA. A simple mathematical model of the myogenic response based on experimental parameters was implemented. Our findings show that the myogenic response is completely preserved in the AA of the Cx40KO and if anything, the stress sensitivity of the smooth muscle cell in the vascular wall is increased rather than reduced as compared to the WT. These findings are compatible with the view of the myogenic response being primarily a local response to the local transmural pressure.

## Introduction

Renal autoregulation aims at maintaining a near constant renal blood flow (RBF) and glomerular filtration rate (GFR) during acute changes in renal perfusion pressure (RPP) (Shipley and Study 1951). Increases in RPP increase renal vascular resistance mainly in the afferent arteriole (AA) proximal to the glomerular capillaries (Carmines et al. 1990) although larger preglomerular vessels also constrict (Sanchez-Ferrer et al. 1989). Renal autoregulation primarily consists of the myogenic response and the tubuloglomerular feedback mechanism (TGF). Whereas the myogenic response is believed to be the main vasomotor mechanism in the proximal part of the AA, the TGF mechanism dominates the part closest to the glomerulus (Casellas and Moore 1990).

Myogenic tone depends on transmural pressure exposing the wall smooth muscle cell to a certain circumferential stress. The latter is likely the variable sensed by the wall (Davis and Hill 1999; Carlson and Secomb 2005). At equilibrium circumferential stress is proportional to both pressure and inner radius and inversely proportional to wall thickness. An increase in pressure therefore causes a parallel increase in the stress which in turn is partially offset by myogenic contraction that reduces radius and increases wall thickness. This negative feedback loop dynamically regulates vessel diameter in the renal arteriolar tree. The myogenic response counteracts the change in flow that would otherwise follow fluctuations in RPP although the myogenic mechanism *per se* is not sensitive to flow (Davis 2012).

The TGF mechanism affects the distal part of the AA resulting in local vasoconstriction. It can be measured several hundred *μ*m away from the juxtaglomerular site where it is elicited (Chen et al. 1995; Wagner et al. 1997). Thus, TGF-induced vasoconstriction can travel upstream in the arteriolar wall probably as an electrical signal, a vascular conducted response. The latter is believed to proceed through gap junctions (Peti-Peterdi 2006; Just et al. 2009; Sorensen et al. 2012); aggregations of pore-forming proteins, built from connexins (Cx), coupling the cytoplasm of two adjacent cells. In the renal vasculature Cx37, Cx40, and Cx43 are expressed primarily in endothelial cells and Cx45 primarily in the smooth muscle cell (SMC) (Kruger et al. 2000; Hanner et al. 2008). Also the myogenic response may have a conducted component (Rivers 1995) and inhibition of SMC gap junctions in cerebral and mesenteric arterioles seems to reduce the myogenic response (Lagaud et al. 2002; Earley et al. 2004).

Cx40 is the main Cx expressed in the juxtaglomerular apparatus where the TGF signaling takes place (Zhang and Hill 2005; Just et al. 2009). Consequently, Cx40 knockout (Cx40KO) mice have a defective TGF mechanism, corresponding functionally to papillectomy (Sorensen et al. 2012). Preglomerular arterioles isolated from Cx40KO mice are unable to conduct a Ca^2+^ signal elicited by electrical stimulation (Sorensen et al. 2012). The lack of Cx40 also results in increased renin release and hypertension in these mice (Wagner et al. 2007).

The aim of this study was to characterize the myogenic properties of the AA in situ in wild-type (WT) and Cx40KO mice. We hypothesized that absence of endothelial Cx40 would not *per se* affect myogenic properties. As Cx40KO mice lack a functional TGF mechanism we tested contractility of the Cx40KO AA against a group of papillectomized WT animals. As Cx40KO mice are hypertensive, interpretation of the results was expected to be complicated by structural remodeling of the AA. In order to better quantify the results, data were therefore fed into a simple mathematical model quantitatively describing the myogenic response in both wild-type and Cx40 KO mice.

Our findings show that the myogenic response is completely preserved in the AA of the Cx40KO and, if anything, the stress sensitivity of the wall smooth muscle cell is increased rather than reduced as compared to the WT. We also find that the Cx40KO shows inward remodeling of the AA likely of the same eutrophic kind as found in other hypertension models.

## Experimental Methods

### Animals

All procedures were approved by the Danish National Animal Experiments Inspectorate. Animals were kept in the animal facility on a 12–12 h day–night schedule and received tap water and standard chow ad libitum.

Heterozygous Connexin 40 knockout animals for breeding were purchased from European Mouse Mutant Archive (Infrafrontier.eu; Munich, Germany). These animals were of a mixed C57Bl/6;129P2 background. Animals were genotyped from tail tip DNA using the DirectPCR kit (Viagen Biotech, LA). Wild-type (WT) animals had the same genetic background. Kidneys from 26 adult mice were used (WT: *N* = 16; Cx40−/− *N* = 10). An additional five pure C57Bl/6 mice (Taconic) were used. Ages ranged from 3 to 6 months.

#### In vitro perfused juxtamedullary nephron technique

Experiments were conducted according to the blood-perfused juxtamedullary nephron technique (Casellas and Navar 1984) adapted to mice (Harrison-Bernard et al. 2003). Briefly, pentobarbital (SAD, Denmark, 50 mg/kg i.p.) anesthetized mice had a cannula placed in the abdominal aorta below the renal arteries. The cannula system includes a blunted needle for introduction into the renal artery and two lines for perfusion and measurement of perfusion pressure. The kidney was immediately perfused with Tyrode buffer (in mMOL/l: NaCl: 136.9; NaH_2_PO_4_: 0.42; NaHCO_3_: 11.9; KCl: 2.7; MgCl_2_: 2.2; d-glucose: 5.6; CaCl_2_: 1.8) containing 5% bovine serum albumin (ICPbio International Ltd, Auckland, New Zealand) and an amino acid mixture (Sigma-Aldrich, Copenhagen, Denmark); pH 7.4. The kidneys were excised and the cannula was advanced into the left renal artery. A longitudinal slice was made along the kidney to expose the papilla without damaging it. The papilla was reflected back to reveal the inner cortical surface. Venous and connective tissue on the cortical surface was cut open to gain access to the renal vasculature.

Larger arteries were ligated (10.0 sutures; Ethilon, Norderstedt, Germany) restricting perfusion to juxtamedullary AA's of the inner cortical surface.

Kidneys were perfused with the Tyrode/5% albumin solution under which the AA has been shown to preserve a normal autoregulatory response (Sanchez-Ferrer et al. 1989; Carmines and Inscho 1992; Imig et al. 1993). RPP could then be raised to 195 mm Hg while maintaining high visibility of the AA. If perfused with blood, visibility is lost at high pressure due to diffuse bleeding from the cut veins.

The preparation was viewed and recorded using an Olympus BX50WI microscope with a PixelFly digital 12 bit CCD camera using the CamWare software (PCO, Kelheim, Germany). Renal perfusion pressure readings were acquired with a PowerLab/8SP data acquisition system (ADinstruments, Colorado Springs, CO). During the experiment, the kidney was superfused with warmed (37°C) Tyrode's solution containing 1% albumin.

#### Experimental protocol

Afferent arteriolar diameter was measured ~100 *μ*m upstream from the glomerulus. Perfusion was initiated at 95 mm Hg followed by equilibration for 15 min. RPP was then increased in steps of 20 mm Hg up to 195 mm Hg. Each pressure step lasted 3 min. Subsequently the perfusion solution was changed to contain 10 *μ*mol/L nifedipine or 0.1 mmol/L papaverine and the pressure steps were repeated. One WT group was papillectomized to interrupt flow in the loop of Henle whereby TGF is abolished (Sanchez-Ferrer et al. 1989; Takenaka et al. 1994). Changes in afferent arteriolar diameter in response pressure changes are hereafter caused solely by the myogenic response enabling assessment of the relative contributions from TGF and myogenic tone. After the first set of pressure steps the perfusion solution was changed to contain 10 *μ*mol/L nifedipine and the pressure steps were repeated.

To obtain parameter input values for the computational model, diameters were measured in two preparations at 0 mm Hg (WT animals, vasculature relaxed with nifedipine after papillectomy). Similarly, wall thickness was measured (WT: *n* = 10; Cx40KO *n* = 10), but in this case at 95 mm Hg perfusion pressure (active vessels).

*Animal groups:* Six groups of experiments were performed: group 1: kidneys from WT mice with/without 10 *μ*mol/L nifedipine (*n* = 5); group 2: kidneys from WT mice with/without 1 mmol/L papaverine to evaluate maximal dilatation (*n* = 5). No differences were found between these two groups, neither in baseline diameter, nor in their passive or active response to pressure changes (see Table2 for details). Consequently, they were pooled and are hereafter referred to as WT mice (*n* = 10). Group 3: kidneys from commercially available C57Bl/6 mice with/without 10 *μ*mol/L nifedipine, included to evaluate strain variation (*n* = 5). Group 4: papillectomized kidneys from WT mice with/without 10 *μ*mol/L nifedipine, for evaluation of contractile properties of the AA without contribution from the TGF mechanism (*n* = 6). Group 5: kidneys from Cx40KO mice with/without 10 *μ*mol/L nifedipine (*n* = 5) and group 6: kidneys from Cx40KO mice with/without 1 mmol/L papaverine (*n* = 5). As also the latter two groups were similar in all aspects (please see Table2) they were pooled and are hereafter collectively referred to as Cx40KO mice (*n* = 10). In all groups RPP was increased from 95 mm Hg to 195 mm Hg in steps of 20 mm Hg. In the following “passive” and “active” states refer to perfusion with and without vasodilator, respectively.

#### Data analysis

Afferent arteriolar diameter was measured off-line using ImageJ (NIH, Bethesda, MD). Arteriolar edges were tracked and diameter measured manually every 10 sec throughout the experiment. Results are presented as the mean of the last minute of every pressure step (*μ*m) ±SEM.

For statistical analysis Sigmaplot software (SyStat Software Inc., San Jose, CA) was used. Changes within groups were analyzed using paired Student's t-test or a two-way ANOVA with repeated measurements. Changes between groups were analyzed using unpaired Student's *t*-test or one-way ANOVA with repeated measurements. A *P*-value <0.05 was considered significant.

### Computational Methods

Only a segment of the mid to distal part of the AA was modeled explicitly. All upstream (i.e., until and including the renal artery) and downstream renal vasculture (i.e., including the glomerulus and until and including the renal vein) only enter the simulations through their effect on the local pressure in the modeled segment (please see below). To simulate the myogenic response we apply a modified version of a previously published general vessel wall model (Jacobsen et al. 2008) and adapted the constants to those used in a model of the rat AA (Feldberg et al. 1995). Structural constants (estimated radius and relative wall thickness of the completely relaxed vessel at 0 kPa transmural pressure) were based on the present measurements.

### The Model

#### Model of the vascular wall

In brief (for details please see (Feldberg et al. 1995; Jacobsen et al. 2008)) the wall is modeled as consisting of passive elastic material (collagen and elastin) as well as an active contractile component. These components are arranged in parallel and the total stress is the sum of their individual contributions.

During contraction or dilatation of the vessel, the trans-sectional area of the wall (i.e., the area of the tissue ring when the vessel is cut perpendicular to its length axis) is conserved. Independently of the level of contraction at a given moment, and with *i* and *o* referring to inner and outer radii of the vessel, respectively, the inwardly directed pressure, *P*, generated by the vascular wall (equal to the transmural pressure at equilibrium), is given by Laplace's law:

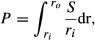
1

where *S* is the Cauchy stress, which can further be expressed in terms of the active and passive stress contributions from each of the thin sections into which the tunica media is divided during numerical integration of Eq. 1 (Jacobsen et al. 2008).

#### Circumferential wall stress and activation of the vascular wall

At equilibrium the average circumferential stress, 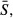 in the wall is given by the following equation:

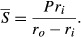
2

**Figure 1:**
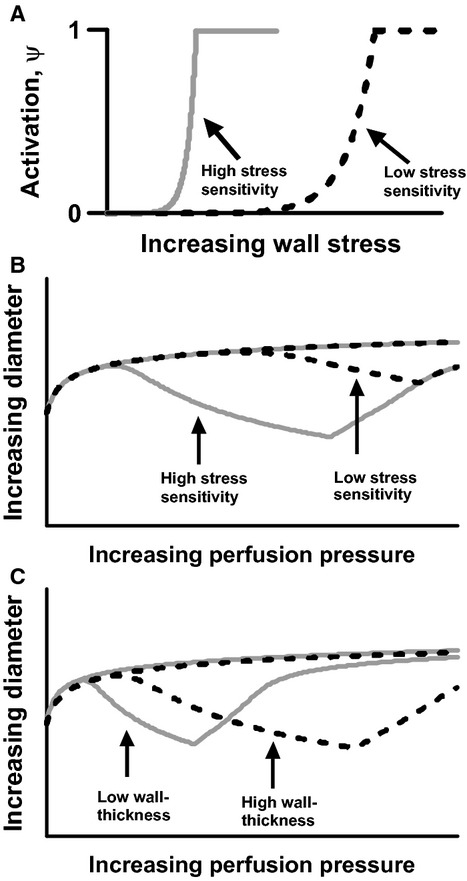
Theoretical examples of (A) activation as a function of increasing wall stress at high (gray) and low (black) stress sensitivity of the vascular wall. (B) Diameter as a function of increasing perfusion pressure at high (gray) and low (black) stress sensitivity. (C) Diameter as a function of increasing perfusion pressure at low (gray) and high (black) wall thickness.

Activation, *ψ*, of the SMC contractile machinery causes active force development. Activation is normalized to lie between 0 (complete relaxation), and 1 (maximal activation). In the present case, we only consider the myogenic response, hence at steady state 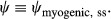 The myogenic response is assumed to be a direct function of the average circumferential wall stress:

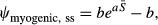
3

with 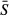 given in kPa and *b* is a constant that scales 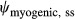 to the physiologically relevant stress range (same value in all cases, see Table1). We denote the exponent *a* “stress sensitivity”. Values for *a* that fit to the experimental curves for each group of vessels are given in the results section. Figure1 panel A shows two examples of activation as a function of wall stress. There are no differences between the simulations except for the stress sensitivity. In case of high sensitivity (*a *=* *0.3, gray curve) activation increases earlier and more steeply as compared to the situation with low sensitivity (*a* = 0.1, dashed curve). The corresponding pressure–diameter curves are shown in Figure1B. High stress sensitivity causes stronger contraction at lower pressures (gray curve) as compared to the situation with low stress sensitivity (dashed curve). The uppermost curves show the diameter response of the completely relaxed vessel.

**Table 1:** Parameters included in the mathematical model of the myogenic response

Strain and intervention	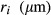 Meas.	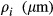 Meas.	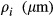 adjusted	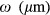 Meas.	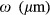 Adjusted	wta 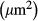	*η* (no unit)
WT (active)	22/2	x	8.0	x	2.33	178.5	1.37
WT (passive)	21.5/2	x	8.0	3.5	2.33	178.5	1.37
WT Papillect. (active)	26.5/2	x	8.0	x	2.33	178.5	1.37
WT Papillect. (Passive)	27.7/2	7.2	8.0	x	2.33	178.5	1.37
KO (active)	19/2	x	6.9	x	2.66	181.5	1.49
KO (passive)	19/2	x	6.9	4.0	2.66	181.5	1.49

*r*_*i*_ (internal radius at estimated 66 mmHg (perfusion pressure 95 mmHg)), *ρ*_*i*_ (internal radius in fully relaxed vessel at 0 kPa transmural pressure), 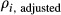 (internal radius in fully relaxed vessel at 0 kPa transmural pressure, without folding of any layer), *ω* (wall thickness at estimated 66 mmHg (perfusion pressure 95 mmHg)), *ω*_adjusted_ (wall thickness adjusted to only include the contractile part (tunica media) at estimated 66 mmHg), *wta* (transsectional area based on adjusted *ω*_adjusted_), *η* (external radius divided by internal radius in fully relaxed vessel at 0 kPa transmural pressure. Relaxed external radius was calculated based on wta and adjusted *ρ*_*i*_) and x (value not measured).

#### Numerical methods and program flow

The active internal radius of the vessel was found by solving (iteratively) the expression:

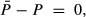
4

where 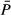 is the transmural pressure and *P* is the inwardly directed pressure generated by the vascular wall. This is possible since, according to Eq. 1, *P* is a function of *r*_*i*_

#### Convergence criterium and implementation

With *δ* being the change in the value of *r*_*i*_ between iterations *n* and *n *+* *1, the criterion was that:

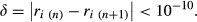
5

In a given simulation this criterion was fulfilled for at all pressure levels.

The model was implemented in c (ANSI C standard) and run in Microsoft Visual Studio on an ordinary desktop computer.

#### Assignment of values to model parameters

All parameter values described in the following are found in Table1. To simulate passive- and active pressure–diameter curves, internal vessel radius (*ρ*_*i*_) and relative wall thickness of the fully relaxed vessel at 0 mmHg transmural pressure 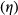 are needed as input parameters. The radius measured under these conditions (please see experimental methods section) is on the order of 70% of the relaxed vessel radius measured at 95 mm Hg perfusion pressure. Reducing the pressure to 0 kPa may cause some compression of the vessel from the surrounding tissue or from the outermost layers of the wall itself. In vessels with an internal elastic lamina (IEL) this may appear as folding of the IEL. In the first-order rat cremaster arteriole the internal circumference measured by tracing the folds is 10–25% larger than internal circumference measured not considering these folds (Bakker et al. 2003). We therefore used the value 8.0 microns instead 7.2 microns (the average of three measurements) as parameter value for *ρ*_*i*_ in the WT animals. As the measured radius (both active and passive) for the KO animals are smaller compared to the WT animals, *ρ*_*i*_ for the KO animals were reduced by the same proportion (0.86) giving a value of *ρ*_*i*_ = 6.9 *μ*m.

The relative wall thickness, *η* (here defined as outer radius divided by inner radius in the relaxed vessel at 0 mmHg) was estimated as follows: Wall thickness was measured at 95 mm Hg (3.49 ± 0.12 *μ*m in WT, 4.02 ± 0.33 *μ*m in Cx40−/−, difference ns). As the vessel wall model only considers the tunica media (contractile) and as other structures (intima, IEL, adventitia) take op part of the wall in the living vessel (see e.g., (Brekke et al. 2002)), the measured wall thickness at 95 mmHg was reduced by 1/3 in order to represent the tunica media only. The wall transsectional area, *wta*,  was calculated as: 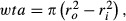 where *r*_*o*_and *r*_*i*_ refer to outer and inner radius, respectively, at 95 mmHg. Assuming that *wta* remain approximately constant as the vessels changes diameter, *η* was found as:

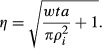
6

Figure1C illustrates how *η* influences the pressure–diameter curves in two vessels that are otherwise identical including identical stress sensitivity (*a* = 0.3 in both cases). When the wall is thin relative to lumen-size (*η* = 1.2,  gray curve), wall-stress and, consequently activation, will increase rapidly following an increase in pressure and the vessel will contract more at a given pressure level as compared to the situation with a relatively thicker wall (*η* = 1.4,  dashed curve). A relatively thicker wall allows the vessel to autoregulate over a broader pressure interval (the sharp turn on the curve corresponds to reaching *ψ* = 1 after which the vessels experience forced dilatation).

#### Estimation of the pressure in the mid to distal part of the afferent arteriole compared to the perfusion pressure

A certain decline in pressure takes place from the renal artery and downstream to the distal part of the AA where the measurements are made. On the basis of data from (Casellas and Moore 1993) giving the pressure in the mid to distal part of the AA as a function of renal perfusion pressure (RPP), we estimated that the RPP interval of the present measurements: 95–195 mmHg = 12.66–26 kPa corresponds to approximately 62–95 mmHg = 8–12.5 kPa actually experienced in the mid to distal part of the AA. This corresponds to the region between the vertical bars on the simulated pressure–diameter curves.

## Results

### Baseline diameter

Active afferent arteriolar diameter at 95 mm Hg (baseline) in WT and Cx40KO mice was significantly different (WT: 22.2 ± 0.8 *μ*m; Cx40KO: 19.1 ± 0.8 *μ*m; *P* < 0.01). Passive baseline diameter was not significantly different from active baseline diameter in either of the groups (WT: 21.6 ± 1.1 *μ*m; Cx40 KO: 19.2 ± 0.8 *μ*m). Active baseline diameter in the papillectomy group was 25.5 ± 1.0 *μ*m. The diameter was not significantly larger in the corresponding passive state (26.5 ± 1.2 *μ*m). Papillectomy significantly increased baseline diameter as compared to the WT-group both in the passive (*P* < 0.05) and in the active state (*P* < 0.01, please see discussion).

### Afferent autoregulation

#### WT mice

**Figure 2:**
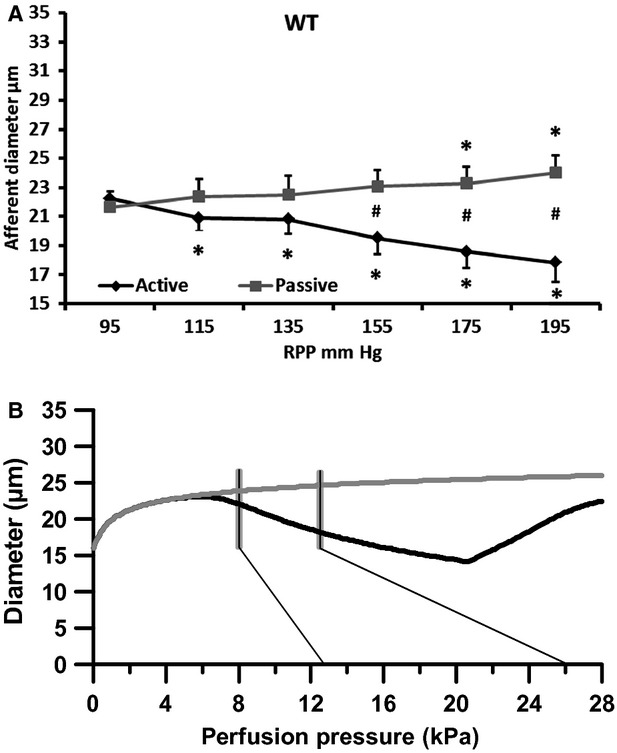
Changes in afferent arteriolar diameter in wild-type mice at increasing renal perfusion pressures during perfusion without (black, active curve) and during perfusion with a vasodilator (nifedipine/papaverine; gray, passive curve). (A) experimental data. (B) data from the mathematical model. Thin black lines show the pressure range (95–195 mm Hg) measured in the renal artery. Gray vertical lines indicate the estimated pressure range found in the mid to late AA. *P < 0.05 versus 95 mm Hg. #*P* < 0.05 versus active.

In WT mice acute step increases in RPP from 95 mm Hg to 195 mm Hg reduced active afferent arteriolar diameter significantly compared to baseline diameter at 95 mm Hg. The diameter decreased from 22.2 ± 0.8 *μ*m to 17.8 ± 1.3 *μ*m (Fig.2A, black curve; *n* = 10). Linear regression analysis of the individual curves revealed an overall slope of −0.048 ± 0.010 *μ*m/mm Hg suggesting a significant autoregulation.

An RPP increase from 95 mm Hg to 195 mm Hg in the passive WT group increased afferent diameter significantly from 21.1 ± 1.1 to 24.0 ± 1.2 *μ*m (Fig.2A, gray curve). The overall slope of the passive curve was 0.022 ± 0.004 *μ*m/mm Hg, which was significantly different from the slope of the active curve (Table2; *P* < 0.01). Figure2B shows the corresponding simulated curves. The vertical lines indicate the pressure interval we estimate is found in the mid to distal part of the AA, when increasing the RPP over the experimental range given in Figure2A. This range (95 – 195 mm Hg) is indicated with the thin lines in 2B. Using the structural data shown in Table1 (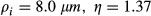) a reasonable fit (black curve) to the active experimental curve is found using a stress sensitivity of *a* = 0.23. Also the simulated passive curve shows slight dilation over the pressure range.

**Table 2:** Slopes of the curves from the six experimental groups and the pooled WT and Cx40KO groups. Bold indicate slope on curves mentioned in Results. Slopes were measured before (active) and after (passive) perfusion with nifedipine or papaverine. Values are mean ± SEM. Differences within groups were found using a paired Student's t-test. Differences between groups were found using a one-way ANOVA followed by Student–Newman–Keul test

	Slope active	Slope passive	Active versus passive	Active	Passive
1. WT ± nif	−0.055 ± 0.016	0.022 ± 0.005	<0.01		
2. WT ± papaverin	−0.037 ± 0.007	0.021 ± 0.006	<0.05		
**WT pooled**	−**0.048 ± 0.010**	**0.022 ± 0.004**	**<0.01**	**versus KO <0.05**	**versus KO NS**
**3. C57Bl6 ± nif**	−**0.032 ± 0.010**	**0.011 ± 0.05**	**<0.01**		
**4. WT papillec ± nif**	−**0.017 ± 0.011**	**0.018 ± 0.006**	**<0.05**	**versus WT <0.05**	**versus WT NS**
5. KO ± nif	−0.018 ± 0.008	0.016 ± 0.007	NS (0.06)		
6. KO ± papaverin	−0.012 ± 0.003	0.016 ± 0.005	<0.05		
**KO pooled**	−**0.016 ± 0.005**	**0.016 ± 0.004**	**<0.01**	**versus papillec NS**	**versus papillec NS**

#### C57Bl/6 mice

To evaluate any effect of the mixed genetic background of the mice used in this study we also used commercially available C57Bl/6 mice. We compared active and passive baseline diameters as well as active and passive responses to acute step-wise increases in RPP from 95 mm Hg to 195 (*n* = 5). In no case were the results significantly different from those found in the WT group (please see Table2), showing that our WT mice are comparable to commercially available C57Bl/6.

#### Papillectomized WT mice

**Figure 3:**
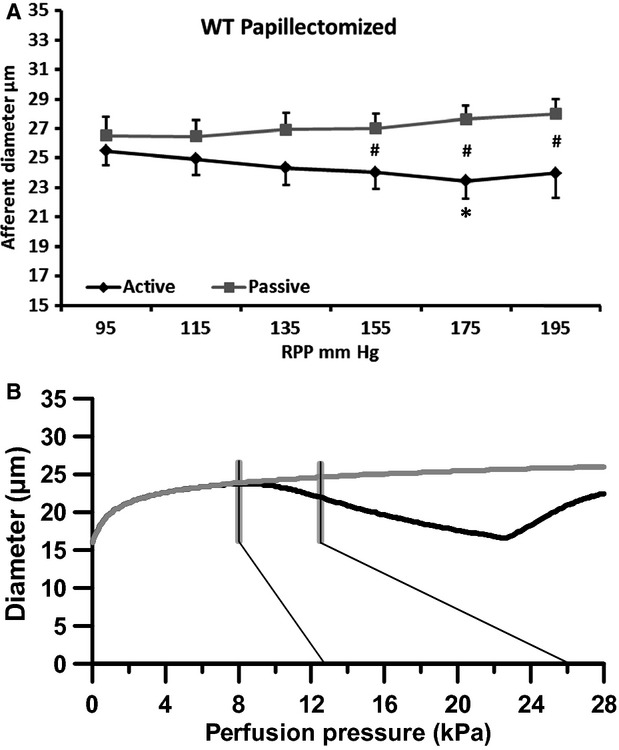
Changes in afferent arteriolar diameter in papillectomized wild-type mice at increasing renal perfusion pressures during perfusion without (black, active curve) and during perfusion with a vasodilator (nifedipine; gray, passive curve). (A) experimental data. (B) data from the mathematical model. Thin black lines show the pressure range (95–195 mm Hg) measured in the renal artery. Gray vertical lines indicate the estimated pressure range found in the mid to late AA. *P < 0.05 versus 95 mm Hg. #*P* < 0.05 versus papillectomy, active.

In papillectomized kidneys from WT mice (group 4) the afferent autoregulatory response is driven by the myogenic response alone as papillectomy removes the TGF response. In this group the pressure steps from 95 mm Hg to 195 mm Hg significantly reduced active afferent arteriolar diameter only at 175 mm Hg (Fig.3A black curve). However, the overall slope of the autoregulation curve was −0.017 *μ*m/mm Hg ±0.011 indicating that the myogenic response is still active and reduces afferent diameter in response to pressure increases. The slope was significantly different from that found in the WT group (see Table2) suggesting reduced afferent autoregulatory capacity. In the passive papillectomized group (10 *μ*mol/L nifedipine) afferent diameter increased from 26.5 ± 1.2 *μ*m at 95 mm Hg to 28.0 ± 1.1 *μ*m at 195 mm Hg (Fig.3A, gray curve). The slope of the passive curve was 0.018 ± 0.006 *μ*m/mm Hg which was significantly different from the active curve (*P* < 0.05), but not different from the passive curve in the WT group (Table2). Figure3B shows the corresponding simulated curves. Although the starting diameter for the papillectomized WT animals appears larger compared to nonpapillectomized WT animals (compare Fig.2A and 3A), the same structural values were used in the simulations (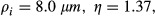 please see discussion for this issue). Hence, the simulated curves do not reach the same absolute radii, but mimics only radius-changes as pressure is increased. Again vertical bars indicate the pressure range to which the AA is assumed to be exposed. The modest reduction in diameter of the active vessels seen in Figure3A (black curve) is mimicked using a stress sensitivity of *a* = 0.16. The passive curve (gray) is identical to that of Figure2B dilating slightly as pressure increases as found also in the experimental case (Fig.3A, gray curve).

#### Cx40 KO mice

**Figure 4:**
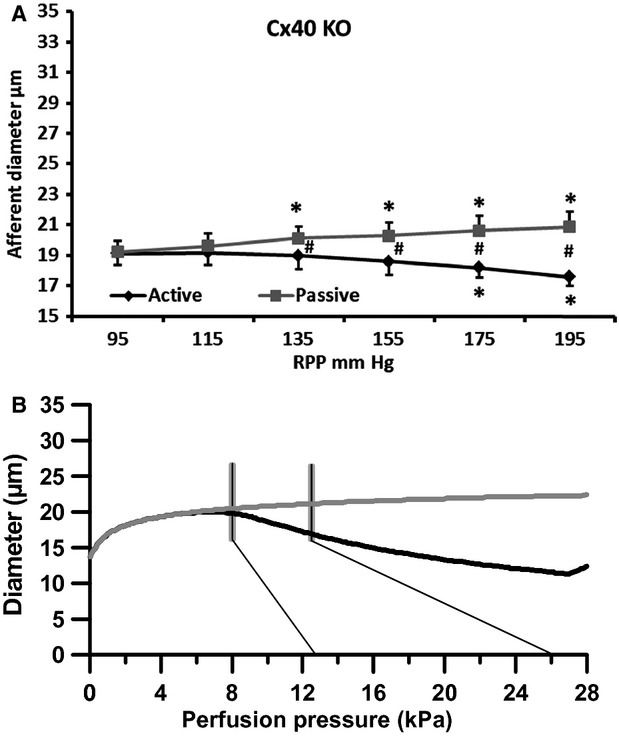
Changes in afferent arteriolar diameter in Cx40 knockout mice at increasing renal perfusion pressures during perfusion without (black, active curve) and during perfusion with a vasodilator (nifedipine/papaverine; gray, passive curve). (A) experimental data. (B) data from the mathematical model. Thin black lines show the pressure range (95–195 mm Hg) measured in the renal artery. Gray vertical lines indicate the estimated pressure range found in the mid to late AA. *P < 0.05 versus 95 mm Hg. #*P* < 0.05 versus active.

In the kidneys from Cx40 KO mice the pressure steps from 95 mm Hg to 195 mm Hg induced a significant afferent diameter reduction from 19.1 ± 0.8 *μ*m to 17.6 ± 0.6 *μ*m (Fig.4A, black curve, *n* = 10). The overall slope was −0.016 ± 0.005 *μ*m/mm Hg which was significantly different from the slope found in the WT group, but not different from the slope found in the papillectomized group (Table2). After perfusion with the vasodilators the afferent diameter increased significantly when RPP was increased from 95 mm Hg to 195 mm Hg (Fig.4A, gray curve). The slope of the passive curve was 0.016 ± 0.004 *μ*m/mm Hg which was significantly different from the active curve (*P* < 0.01), but not significantly different from the slope of the passive curve seen in the WT group (Table2).

Comparing the passive curves from the WT and the Cx40KO groups showed that, similar to what was observed at 95 mm Hg, the diameter measured at 195 mm Hg was significantly smaller in Cx40KO (20.9 ± 1.0 *μ*m) compared to WT (24.0 ± 1.0 *μ*m; *P* < 0.05).

Figure4B shows the active and passive simulated Cx40KO curves using the structural parameters given in Table1 (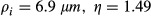). As before vertical bars indicate the pressure range to which the AA is presumably exposed. The smaller starting diameter causes a downward shift in the passive curve as compared to the same curve of Figure2B, but dilation is still observed as pressure increases. To mimic the response of the active curve it is necessary to use a stress sensitivity of *a* = 0.26. Hence, because the wall is relatively thicker in these vessels it must be more sensitive to stress to give the quite strong response seen in the experimental case (c.f. Fig.1B, C).

## Discussion

The present experiments investigate whether loss of Cx40 affect the renal afferent myogenic response. The results show that as opposed to the TGF the myogenic response measured in the AA is not dependent on expression of Cx40. If anything, loss of Cx40 causes an increase, rather than a decrease, in sensitivity to circumferential wall stress induced by the transmural pressure difference.

We have previously described a reduced autoregulatory response in AA's during acute pressure increases (up to 155 mm Hg) in the Cx40KO mouse (Sorensen et al. 2012). Also, we previously found that the TGF in medullary nephrons of the Cx40KO is not functional (Sorensen et al. 2012). The finding in this study of a similar appearance of the active response in papillectomized WT and in Cx40KO animals is indicative of an absent TGF in the Cx40KO. We therefore assume that the autoregulatory response of the Cx40KO medullary AA is of myogenic origin although Oppermann et al. (2013) reported residual TGF activity in some, though not all, Cx40KO AA's. These vessels were, however, of cortical, not medullary origin.

In the present experiments, we increased RPP to 195 mm Hg to ensure that our previous results were not influenced by the hypertension found in the Cx40KO mice through a resetting of the renal autoregulation to higher pressure levels. Such resetting has been found in other hypertensive models (Iversen and Ofstad 1984). AA diameter in spontaneously hypertensive rats showed reduction in diameter to an increase in pressure also above 120 mm Hg (Hayashi et al. 1989; Gonzalez et al. 1994). We did find a significant reduction in afferent arteriolar diameter at RPPs above 155 mm Hg. However, the slope of the autoregulation curve in the Cx40KO mice was significantly less steep as compared to the slope in the WT mice but it was similar to the slope found in the papillectomized WT mice. Thus, also at higher RPP the autoregulatory response in the AA is reduced when Cx40 is lacking and is similar to the response found in papillectomized WT mice. Hence, the myogenic response in Cx40 KO mice is similar, although with a changed stress sensitivity, to the myogenic response in WT mice.

One important difference from our previous experiments is the use of cell-free perfusion buffer. Renal vascular resistance is lower in this situation (Carmines and Inscho 1992; Imig et al. 1993) and a general reduction in vessel tone is observed in the present experiments. The increased afferent baseline diameter reduces the effect of perfusion with a vasodilator. This has also been seen in hydronephrotic kidneys perfused with cell-free perfusate where increasing doses of nifedipine did not alter baseline afferent arteriolar diameter (Hayashi et al. 1989). Also in the present experiments we found no difference between active and passive diameter at 95 mm Hg perfusion pressure. A general reduction in tone, however, does not change the conclusions as all groups were exposed to the same cell-free conditions.

The myogenic response has been shown to spread within the vascular network to remote areas not directly subjected to pressure changes (Rivers 1995). The conducted vasoconstriction in response to pressure increases could be measured more than 500 *μ*m away from the local site. This indicates that pressure-induced resistance changes are communicated along the vascular wall possibly as endothelial depolarization running through gap junctions, but the physiological significance of this phenomenon remains unclear. In the renal vasculature vasoconstrictor responses are known to conduct both upstream and downstream from the site where they are elicited (Steinhausen et al. 1997). One would therefore expect that pressure-induced vasoconstriction would show stronger conduction in a vasculature with better intercellular coupling. We previously showed that focal electrical stimulation in interlobular arteries leads to an intracellular Ca^2+^ increase measurable 500 *μ*m away from the stimulation site. This conduction was significantly decreased in the Cx40KO (Sorensen et al. 2012). The finding of an equal afferent autoregulatory response in papillectomized WT and Cx40KO in this study, however, suggests that Cx40 does not play a central role in the renal myogenic response. This is somewhat in contrast to findings in other vascular beds. In mesenteric and cerebral arteries inhibition of gap junctions significantly reduces myogenic tone (Lagaud et al. 2002; Earley et al. 2004). Cx40 is primarily expressed in endothelial cells, but one study did show Cx40 expression in vascular smooth muscle cells in AA's from mice (Zhang et al. 2006) and inhibition of Cx40 significantly reduces endothelial derived hyperpolarization in the kidney (De Vriese et al. 2002) suggesting that Cx40 is also part of the renal myoendothelial junction. Taken together, however, the present results suggest that although myoendothelial communication may be affected by the absence of Cx40 it has no substantial effect on the reaction of the renal vasculature to pressure changes. This fits with the general concept that the myogenic response is primarily a local response to the local transmural pressure.

On the basis of in vitro measurements in the rat kidney (Casellas and Moore 1993; Imig et al. 1994), we assume in our model that the pressure reaching the distal AA is substantially smaller than the pressure measured in the renal artery. In the juxtamedullary nephron preparation perfused with cell-free buffer Imig et al. measured interlobular artery pressure to 144 mm Hg at a RPP of 160 mm Hg (Imig et al. 1994). However, glomerular capillary pressure was only 52 mm Hg. This demonstrates the presence of a large resistance along the terminal parts or the arteriolar tree, leading to a significantly reduced pressure reaching the distal part of the AA and the glomerulus. In isolated perfused hydronephrotic kidneys, which are also deprived of a TGF response, pressure increases (80–180 mm Hg measured at the level of the renal artery) induced constriction of the middle and distal part of the interlobular artery (ILA) (Hayashi et al. 1992). In the isolated blood perfused juxtamedullary nephron preparation, a significant constriction of the arcuate artery and ILA was also found (Carmines et al. 1990; Casellas and Moore 1993). Due to this autoregulatory contribution of the upstream vascular tree; in our experiments increasing renal perfusion pressure from 95 mm Hg to 195 mm Hg is therefore estimated to correspond to pressures between 60 and 90 mm Hg reaching the distal AA (Casellas and Moore 1993).

To investigate whether the hypertension seen in Cx40 KO affects the structure of the AA's, passive pressure curves were obtained in the presence of nifedipine and papaverine. In all three groups the passive curve was significantly different from the active curve. The slopes of the passive curves were, however, identical between the three groups. Hence, hypertension in Cx40KO mice does not seem to substantially change the ability of the AA to dilate, which is similar to what is observed in the SHR (Hayashi et al. 1989). Although slopes were similar, we found that radii were significantly lower in the Cx40KO. Wall thickness also tended to be larger in the Cx40KO animals although this did not reach statistical significance. This is in agreement to results obtained from the SHR rat where baseline diameters of arcuate arteries, interlobular arteries and AA's were significantly smaller in SHR compared to WKY (Gebremedhin et al. 1990; Hayashi et al. 1992). On calculating the adjusted *wta* this was found to be similar in the two groups. Bearing in mind that the Cx40KO animals are hypertensive with a MAP elevated approximately 25% compared to WT (de Wit et al. 2000), these findings fit well with the general microvascular changes found in hypertension namely a redistribution of the same amount of wall material around a smaller lumen. This process is known as *inward eutrophic remodeling*, which allows the vessel to withstand a larger pressure while experiencing the same amount of circumferential wall stress (Mulvany 1999).

Both active and passive experimental curves appear rather linear compared to what would be expected from the normal active and passive curves from isolated arterioles (Osol and Halpern 1985; Takenaka et al. 1994; Sorensen et al. 2012). This probably has several explanations. First, the lowest RPP used in the experiments (95 mmHg) is already quite large, even when adjusted for the likely upstream pressure decline (to 66 mmHg). Therefore the characteristic shape of the low-pressure part of the myogenic steady-state pressure–diameter curve (Osol and Halpern 1985; Falcone et al. 1991) where the vessel, starting from 0 kPa, initially dilates, is not seen. Second, as assumed in the simulations, resistance of the upstream network (down to the point in the AA where measurements are made) will lower and narrow the pressure interval actually experienced in the AA during the experimental procedure. Although being in the myogenically active pressure range only a part of the curve is therefore seen which may further add its linear appearance. It should be noted that a linear appearance of an active curve (c.f. Fig.3) is not in conflict with a well-developed myogenic response; it requires increasing myogenic tone to maintain a given radius as pressure is increased.

In the simulations we have assumed that the same pressure interval is experienced by the AA in the active and the passive situation. We recognize that this is an approximation and that the AA's in the passive situation may experience a higher pressure for a given RPP. The simulated curves (Figs.4B) show that moving the interval experienced by the AA to the right and even expanding it somewhat would not make a large difference; the curves would still be rather linear in appearance. Another question relates to why little or no difference is seen between the active and the passive curves at the lowest pressure level where one would expect the passive curve to be consistently larger. As indicated by the simulated curves this is potentially explained by the reduced pressure reaching the AA's. This pressure (8–12.5 kPa) is comparable to the RPP on the pressure–diameter curve where the active and passive curves are only beginning to separate (i.e., the point where the vessel is beginning to develop tone).

Rather surprisingly was baseline diameter in the papillectomized series consistently larger, although in both the WT and in the Cx40KO groups there appear to be little or no tone at that pressure. In previous studies papillectomy had little effect on AA diameter (Ikenaga et al. 1996; Sorensen et al. 2012). The reason for our finding is unclear, but we have no reason to believe that the vessels in the papillectomized group should be structurally different. Consequently we have used the same values for *ρ*_*i*_ and *η* as those used for the WT group.

In conclusion, the present results show that the myogenic response of the mouse AA is not affected by the lack of Cx40 expression. The hypertension developed in these animals causes inward remodeling which is likely to be eutrophic in nature and thus similar to what is seen in the SHR and in human essential hypertension. Whereas the overall diameter response of the Cx40KO AA was not different from the papillectomized WT group, the model suggests that there could be an increase in the underlying stress sensitivity of the Cx40KO vascular wall. We also conclude the normal autoregulatory response of the AA, as expected, is the sum contribution from TGF and myogenic contractility, such that AA's from TGF-deficient Cx40KO mice behave similar to those from papillectomized mice.
